# Antioxidants: Terminology, Methods, and Future Considerations

**DOI:** 10.3390/antiox8080297

**Published:** 2019-08-09

**Authors:** Khiena Brainina, Natalia Stozhko, Marina Vidrevich

**Affiliations:** 1 Department of physics and chemistry, Ural State University of Economics, 620144 Ekaterinburg, Russia; 2 Institute of Chemical Technology, Ural Federal University, 620000 Ekaterinburg, Russia

**Keywords:** antioxidants, terminology, methods

## Abstract

Unreliable terminology and incompatible units of antioxidant activity/concentration expression lead to the failure of antioxidant clinical trials, ambiguity of conclusions about the effect of a chosen therapy in medicine and evaluation of food quality, diet, difficulties using information in monitoring the training process in sports, etc. Many different terms (antiradical activity, antioxidant activity, antioxidant capacity, antioxidant power, antioxidant ability) and methods: Trolox equivalent capacity assay (TEAC), Ferric Reducing Antioxidant Power assay (FRAP), Cupric Reducing Antioxidant Capacity assay (CUPRAC), antioxidative activity assay (ABTS), the oxygen radical absorbance capacity (ORAC), and different options of electrochemical ones) proposed for the determination of antioxidants are described. Possible approaches to the development of this field of science and practice are considered.

## 1. Introduction

The 18th International Congress of the International Society of Antioxidants and Health (ISANH), held in Beirut on 3–4 May 2017 [1], focused on addressing the problem of the onset and role of oxidative stress (OS) in very serious diseases (diabetes, cancer, neurodegenerative disorders, infertility, etc.). The main condition for understanding and evaluating the role of OS in pathogenesis is the possibility of its monitoring. The latter is very important both for clinical studies and for the selection of therapeutic agents, their quality control and optimization of the therapy and diet, and for assessing the quality of food, nutrients, cosmetics, and pharmaceuticals. The problems of monitoring are determined by the complexity of the biological matrix and rapid change of its composition after sampling; variety of compounds of different chemical nature, possessing oxidative and antioxidant properties; a short period of life of radical compounds that play a major role in the processes of life support; the absence of a single term and comparable expression units of concentration and antioxidant properties of a compound or a complex of compounds; and use for quantifying oxidative stress of bioorganic molecules with various properties that do not always correctly simulate processes in a living organism [1,2,3,4,5,6]. Thus, the use of stable radicals, including those generated during the analysis, makes the interpretation of the results concerning oxidative stress insufficiently correct, since the nature and chemical transformations of these and natural radicals vary greatly.

As a result, the current situation limits the use of numerous available data [7,8]. The reason for this situation, as follows from the information above and given below, is that the terminology in the area is ambiguous, thermodynamic and kinetic parameters are often mixed, and experimental analytical methods are based on different principles and use different ways of expressing the results. This leads to incompatibility of results, for example, unreasonable interpretation of the results of clinical studies and even skepticism in the importance of developments in the field and refusal of some journals to consider papers on the topic [9].

Antioxidants are the main reactive oxygen species (ROS) and reactive nitrogen species (RNS) scavengers. ROS and RNS are oxidative stress generators [10,11,12]. ROS and RNS include compounds of radical and nonradical nature. Both of them participate in oxidative stress appearance. The former initiate and participate in chain reactions, while the latter are chemical oxidizing agents. Regardless of the mechanism of the processes, the resulting OS is destructive to a living organism. In this regard, it is difficult to overestimate the role of antioxidants, as scavengers of ROS and RNS [13,14,15].

Halliwell and Gutteridge proposed defining an antioxidant as “any substance that, when present at low concentration compared with those of an oxidizable substrate, significantly delays or prevents oxidation of that substrate” [16].

This is a very fast-growing section of chemistry, biochemistry and bioanalysis in particular. A lot of works are published in the field. According to Scopus, the number of papers containing the word “antioxidants” in journals published in 2000–2018 is 301,795.

The aim of the work is a brief analysis of terminology and methods and consideration of methods for analyzing different samples and expressing results, discussing the prerequisites for obtaining comparable results.

## 2. Terminology

The situation as it had developed by 2013 is covered in [17]. It has not undergone significant changes since then, except for the development of new methods, in particular electrochemical ones, which are useful in solving a number of problems mentioned in the work cited above.

It is worth paying attention to the terms ‘antioxidant’ and ‘antiradical’ properties: An antiradical property/activity characterizes the ability of components to react with free radicals, but an antioxidant property/activity represents the ability to inhibit (reduce) all molecules having high Ox/Red potential, which makes them destructive for body structures [18]. This makes the term “antioxidant property” more general, that is, more correct.

Antioxidant properties are proposed to be defined as “antioxidant capacity” [6,19] and “antioxidant activity”, “antioxidant power” [20], “antioxidant ability” [5]. The first one is interpreted as a “measure of the moles of given free radical scavengers by a test solution”.

Sometimes, different meanings are attributed to the terms “antioxidant activity” and “antioxidant capacity”.

**Figure 1:**
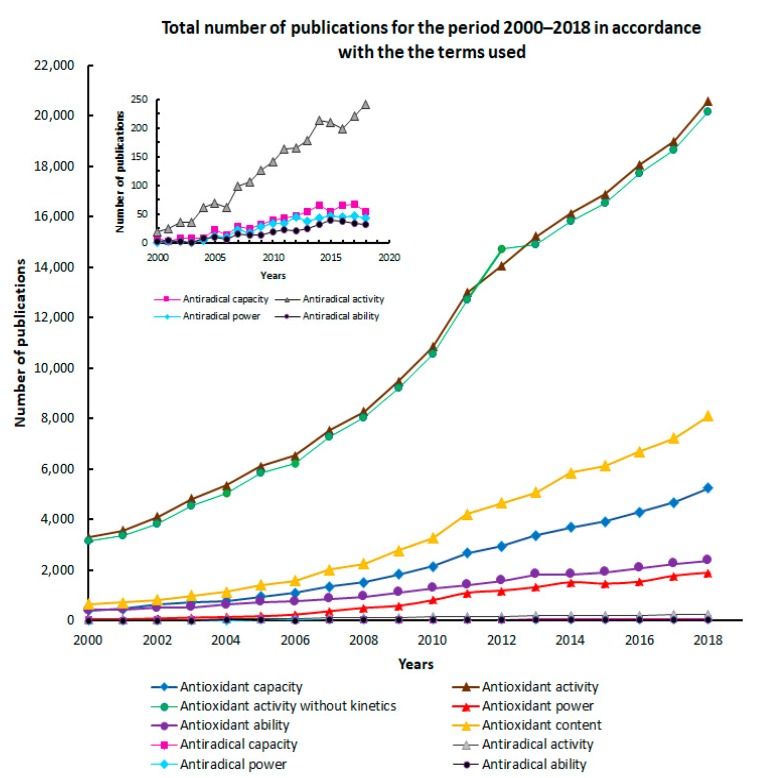
Use of terms in 2000–2018.

There is a mixture of thermodynamic and kinetic concepts: For example, the term antioxidant activity in different works is used as a thermodynamic or kinetic one. In general, it should be used as a thermodynamic term and not as a kinetic one. The term “antioxidant activity” as a kinetic one (Figure 1) is used rarely. The terms “antioxidant power” [20] and “antioxidant ability” [5] do not have a definite interpretation.

The use of these terms in the literature by year (covering the period from 2000 to 2018) can be seen in the diagram presented in Figure 1.

**Figure 2:**
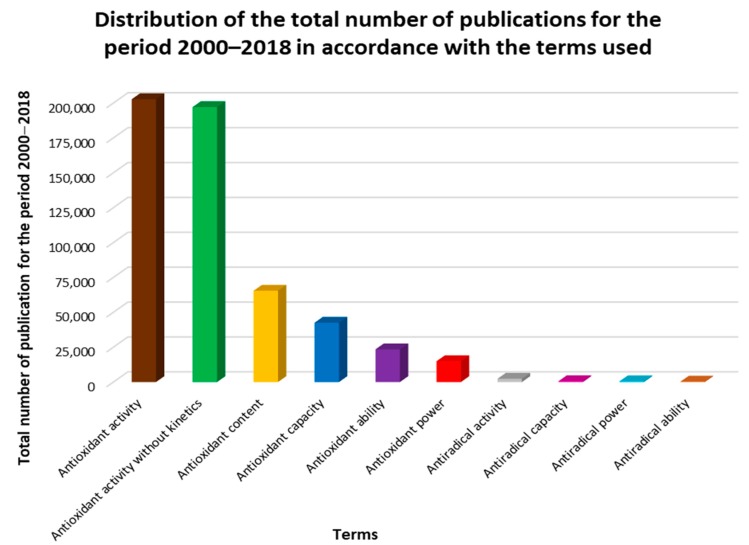
Frequency of terms used in 2000–2018.

The frequency of one or another term application is illustrated by the diagram in Figure 2. The figures show data including yearly distribution of the publication numbers in which the listed terms are used in the title, abstract, and keywords.

It is easy to see that the term “antioxidant activity” is the most commonly used. This seems to be natural, since this term gives direct information about the total concentration of antioxidants/oxidants in the sample.

We note an important fact: In [17] and a number of other works, the terms antioxidant capacity/activity are not separated.

## 3. Methods

There are two main approaches (and, accordingly, groups of methods) to assessing the oxidative/antioxidant status of an organism. The first is associated with direct determination of the content of individual high-molecular (enzymatic) and low-molecular (glutathione, uric acid, ascorbic acid, tocopherols, polyphenols, carotenoids, retinol, etc.) antioxidants (AO). The second approach is based on an assessment of their integral content. Taking into account a large number of various antioxidant compounds, differences in mechanisms and the possibility of synergism of their action in the body, the second approach should be considered preferable and more informative. A fairly complete description and comparison of the main most popular integral methods: antioxidative activity assay (ABTS), Cupric Reducing Antioxidant Capacity assay (CUPRAC), DPPH, colorimetric *in vitro* assay of phenolic and polyphenolic antioxidants (Folin–Ciocalteu), and Ferric Reducing Antioxidant Power assay (FRAP) are described in original papers [19,20,21] and reviews [22,23,24,25]. It is necessary to note that the use of synthetic free radicals which have nothing in common with free radicals in the human body causes, to a large extent, the weaknesses of the methods. This makes interpretation of the results concerning oxidative stress not quite correct.

Calculations in different methods usually consist of comparisons of the signal of the sample with the signal of the reference material. Antioxidant activity/capacity/ability (different terms are used) is expressed in relative units of trolox, rutin, ascorbic acid or something like this. In some cases, lag-time is measured and compared with the same parameter, obtained in the study of the reference compound taken in a known concentration. Thus, the reference value is taken as a result. Since different substances are used as a standard and the concentration is expressed in different units, for example, in g, comparison of the results is possible only in particular cases, even though the results are expressed in dimensionless units.

The feature of electrochemical methods is that no reference material is needed, as a preliminary built calibration curve or addition method are used. The result is a concentration value. In potentiometry, even a calibration curve is not required. The value of the activity/concentration is obtained immediately as a result of direct measurements carried out in the study of the sample.

Data obtained with the use of different methods are difficult to compare with each other, which makes their interpretation complicated. Thus, the situation is associated with the lack of a unified terminological approach and generally accepted units of measurement; random selection of oxidizing reagents, ambiguity, and incompatibility of results limit the use of numerous available data [9].

Electrochemical methods are more promising for the measurement of integral antioxidant properties because the reaction between active oxygen compounds in aqueous media is accompanied by electron transfer, i.e., they are electrochemical in nature.

The driving force of the reaction is a decrease in the free energy of the system:

Δ*G* = Δ*H* − *T*ΔS.

In the case of an electrochemical reaction:

Δ*G* = *nFE*,

*E* = *E*° + (*RT*/*nF*)*lna*,

*a* = *fc*,

where ∆*G*, ∆*H*, and ∆*S*—change in Gibbs free energy, enthalpy, and entropy in the process, respectively; *E*—electrochemical potential; *E°*—standard electrode potential; *a*—activity; *f*—activity coefficient; and *c*—concentration.

The approach includes two main factors: thermodynamic characteristics of the reaction—the possibility, direction, and degree (equilibrium position) of its course and the concentration of the active component. The kinetics of the reaction is determined by the mechanism, energy barrier, presence of catalysts or inhibitors, temperature, and concentration. The factors listed above are interrelated. When considering the interactions of AO with ROS/RNS, everything must be taken into consideration.

There are different approaches to the interpretation of these interactions and methods for evaluating the effectiveness of AO as components of the body’s antioxidant defense system. Apparently, the main quantitative parameter in evaluating the effectiveness of AO is the content/concentration of AO in the sample, and methods that give direct information about this value should obviously be chosen to monitor AO and assess oxidative stress.

The authors of [26] described in detail the electrochemical methods for determining the integral antioxidant activity/capacity. Taking into account that the aim of the paper is to discuss the situation with terminology, not to consider methods, I am not sure it is worth describing more papers on electrochemical methods here. Examples of using the electrochemical methods, e.g., in the food [27] and wine [28], are presented. In [29], results obtained with the use of cyclic voltammetry (CV) and DPPH assays for the determination of antioxidant capacity are compared. Different directions of electroanalytical chemistry are used: coulometry, voltamperometry in various variants (cyclic, differential-impulse, square wave), amperometry, chronoamperometry, and potentiometry.

Electrochemical methods are based on redox reactions occurring on the electrode surface (voltammetry, amperometry) or in the solution volume (chronoamperometry, potentiometry, coulometry), the result of which is recorded as current or potential [30,31,32,33,34]. Processes involving chemical and electrochemical reactions can be considered as hybrid variants. Usually, mediator systems such as DPPH/DPPH, K_3_[Fe(CN)_6_]/K_4_[Fe(CN)_6_], I_2_/2I^−^, Br_2_/2Br^−^ [25] or a reagent specifically introduced into the test solution [30] are used. Of interest is the contact version of the method [32,33]. The main results obtained by this method are given in [35,36,37,38].

As regards the electrochemical methods:

- Most fully meet the nature of the OS and the action of the body’s antioxidant defense system;
- They are characterized by instrumental and operator simplicity, financial, temporal, and informative efficiency;
- They are easily applied in the clinical laboratory, and on their basis, sensory systems are created for work in onsite and in situ formats.

Electrochemical methods give direct information about the concentration of the analyte. In connection with these, we believe that the term “activity” is preferred. We are convinced that it is precisely this term and not “capacity” that does not have a certain physical meaning, so it is advisable to use it to characterize the antioxidant system. The latter is especially significant when using electrochemical monitoring methods, for example, in [39], because it is the activity that determines directly the potential of the electrode that serves as a source of analytical information.

## 4. Ways of Development and Prospects

The questions considered in the article are fundamental for the development of the antioxidants and oxidative stress science, evaluation of its role in the state of health, and the use of AO in diet and therapy. Taking into account the very significant role of AO in life support, it is crucial to recognize the need of studies focusing on the food–nutrients–health–therapy chain.

Understanding and choosing a treatment strategy, diet, etc. is impossible without the creation of uniform standardized methods and comparable ways of expressing concentration, conducting clinical trials, and making recommendations.

We see no reason for skepticism when considering the informativeness of data on antioxidant/oxidant activity. Rather, the issue is the inconsistency of these data and the lack of standardized analytical methods and clear and comparable ways of expressing measurement results.

Trends and ways of development, in addition to the ones discussed above, include expansion of applications, in particular, in sports, the development of new sensors, for example, wearable, and instruments for onsite and online application.

Monitoring the oxidant/antioxidant state of the media is a tool for the development of a number of areas of science and technology: medicine, sports, food, pharmaceutical, cosmetic, and other technologies. Research in this area plays an important role in the detection of serious diseases in the early stages, the choice of therapy, and the evaluation of its effectiveness.
